# Improvement of Resistive Switching Performance in Sulfur-Doped HfOx-Based RRAM

**DOI:** 10.3390/ma14123330

**Published:** 2021-06-16

**Authors:** Zhenzhong Zhang, Fang Wang, Kai Hu, Yu She, Sannian Song, Zhitang Song, Kailiang Zhang

**Affiliations:** 1 Tianjin Key Laboratory of Film Electronic and Communication Devices, School of Electrical and Electronic Engineering, Tianjin University of Technology, Tianjin 300384, China; zhangzz_1128@163.com (Z.Z.); fwang75@163.com (F.W.); yushe_11@163.com (Y.S.); 2 State Key Laboratory of Functional Materials for Informatics, Shanghai Institute of Micro-System and Information Technology, Chinese Academy of Sciences, Shanghai 200050, China; songsannian@mail.sim.ac.cn (S.S.); ztsong@mail.sim.ac.cn (Z.S.)

**Keywords:** HfOx-based RRAM, sulfur-doping, RS performances improvement, switching mechanism

## Abstract

In order to improve the electrical performance of resistive random access memory (RRAM), sulfur (S)-doping technology for HfOx-based RRAM is systematically investigated in this paper. HfOx films with different S-doping contents are achieved by atmospheric pressure chemical vapor deposition (APCVD) under a series of preparation temperatures. The effect of S on crystallinity, surface topography, element composition of HfOx thin films and resistive switching (RS) performance of HfOx-based devices are discussed. Compared with an undoped device, the V_SET_/V_RESET_ of the S-doped device with optimal S content (~1.66 At.%) is reduced, and the compliance current (Icc) is limited from 1 mA to 100 μA. Moreover, it also has high uniformity of resistance and voltage, stable endurance, good retention characteristics, fast response speed (SET 6.25 μs/RESET 7.50 μs) and low energy consumption (SET 9.08 nJ/RESET 6.72 nJ). Based on X-ray photoelectron spectroscopy (XPS) data and fitting of the high/low resistance state (HRS/LRS) conduction behavior, a switching mechanism is considered to explain the formation and rupture of conductive filaments (CFs) composed of oxygen vacancies in undoped and S-doped HfOx-based devices. Doping by sulfur is proposed to introduce the appropriate concentration oxygen vacancies into HfOx film and suppress the random formation of CFs in HfOx-based device, and thus improve the performance of the TiN/HfOx/ITO device.

## 1. Introduction

Resistive random access memory (RRAM), as the emerging non-volatile memory, has the potential to replace traditional NAND flash [1,2,3]. RRAM has some unique advantages: its simple structure, good scalability, low operating voltages, low energy consumption, fast reading and writing speed, high memory density and excellent compatibility [4,5]. As the excellent resistive medium layers of RRAM, binary-transition metal oxide (B-TMO) materials (HfOx [6], TiO_2_ [7], ZnO [8], ZrO_2_ [9], TaOx [10], WOx [11], etc.) have been objects of research interest [4,12]. Among them, the high-k (~21) HfOx is considered one of the most promising materials for commercial applications due to its overall performances in terms of reliability, including endurance, retention and operation stability, etc. [13,14]. However, it is still difficult to comprehensively improve device performances; for example, its low energy consumption and high cycle-to-cycle uniformity. Researchers have proposed some solutions to solve the existing problems for RRAM: ion doping [9,12,15,16], double-layer structure [17,18,19], electrode engineering [20,21], etc. Among these methods, ion doping is considered an effective method to improve device uniformity, energy consumption and other performances [15,16,22]. There have recent related studies on sulfur (S)-based resistive switching devices. Jang et al. [23] reported the Ag_2_S based RRAM with improved endurance and retention using different electrode materials. Li et al. [24] reported a HfS_2_ film memory device based on density functional theory calculations. Zheng et al. [25] proposed an electrode annealing method to improve the RS properties in PbS micro/nanowire-based devices for RRAM.

In our work, S-doped HfOx RRAM devices are fabricated using atmospheric pressure chemical vapor deposition (APCVD) treatment. The resistive switching performance of the HfOx-based devices are improved and the physical characteristics of the S-doped HfOx films are investigated. The S dopant is suggested to introduce an appropriate concentration of oxygen vacancies into HfOx film and suppress the random formation of CFs in the HfOx-based device.

## 2. Experimental Section

**Figure 1:**
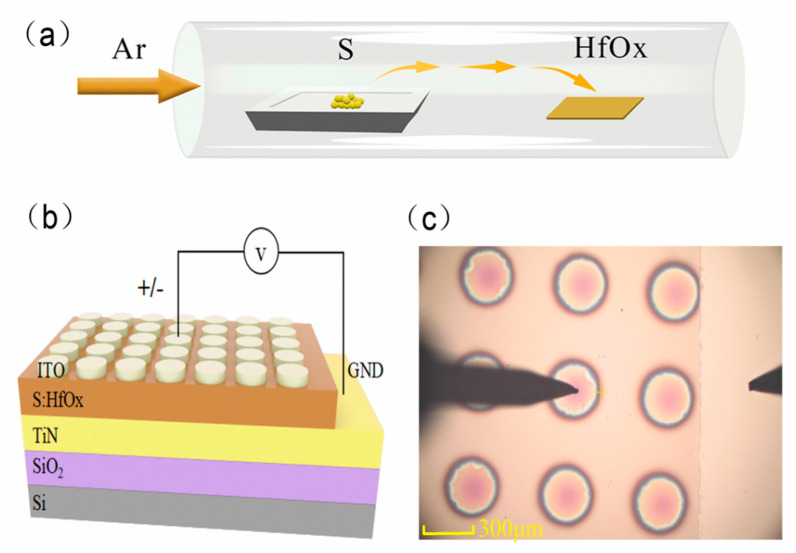
(**a**) Schematic diagram of two-temperature zone tube furnace. (**b**) Structure of TiN/S:HfOx/ITO for the S-doped device (The left probe contacts the ITO electrode and the right probe contacts GND). (**c**) Optical micrograph of the device.

A layer of 50-nm TiN film as the bottom electrode (BE) was first deposited on a cleaned Si substrate by direct current (DC) sputtering with Ti as the metal target (99.99%) under an Ar/N_2_ atmosphere. A 7-nm thick HfOx thin film was sputtered on TiN with a HfO_2_ ceramic target (99.99%) under an Ar/O_2_ atmosphere. Then, the HfOx film was treated by APCVD for 30 min under sulfur atmosphere at different temperatures of 400 °C, 500 °C, 600 °C. The Ar flow rate was maintained at 30 sccm during the whole process, as shown in Figure 1a. After that, a 200-nm thick top electrode (TE) was deposited by the indium tin oxide (ITO) ceramic target (99.99%) with a 300-μm diameter metal mask. Figure 1b,c shows the structure of TiN/S:HfOx/ITO and an optical micrograph of the device, respectively. The chemical compositions of the undoped and S-doped HfOx films were characterized by XPS (Scientific Escalab 250Xi, Waltham, MA, USA). The film morphology was observed via atomic force microscopy (AFM, Bruker Dimension Icon, Santa Clara, CA). The electrical performances of undoped and S-doped HfOx-based devices were measured using a semiconductor parameter analyzer (Agilent B1500A, Santa Clara, CA, USA). For the electronic measurement of samples, a bias voltage was applied to the TE of ITO while the BE of TiN was grounded.

## 3. Results and Discussions

**Figure 2:**
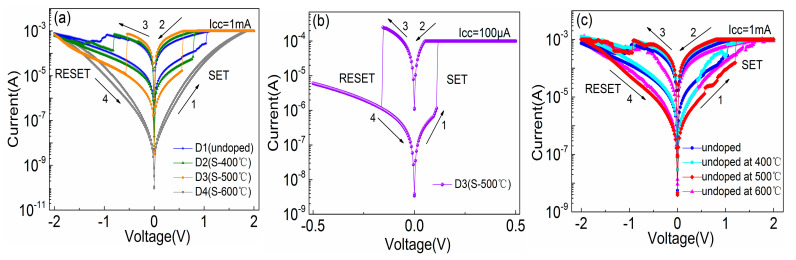
(**a**) Typical bipolar I–V cycles for DC sweep of the devices, (**b**) I–V cycle of D3 device under 100 μA compliance current, (**c**) I–V cycles for the untreated and undoped at different temperatures annealing of the devices.

**Figure 3:**
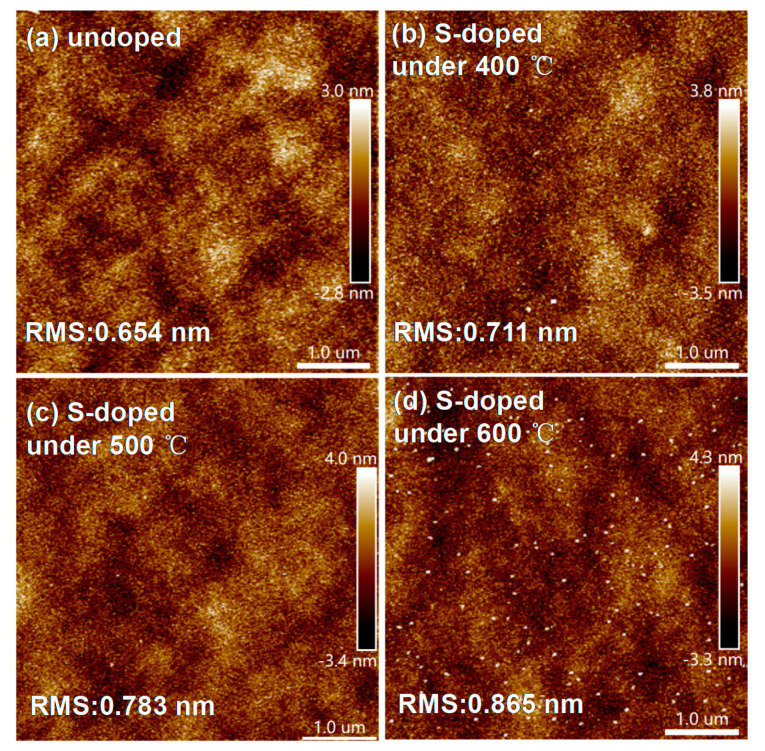
AFM images of the HfOx films of (**a**) undoped and S-doped at (**b**) 400 °C, (**c**) 500 °C, and (**d**) 600 °C.

**Figure 4:**
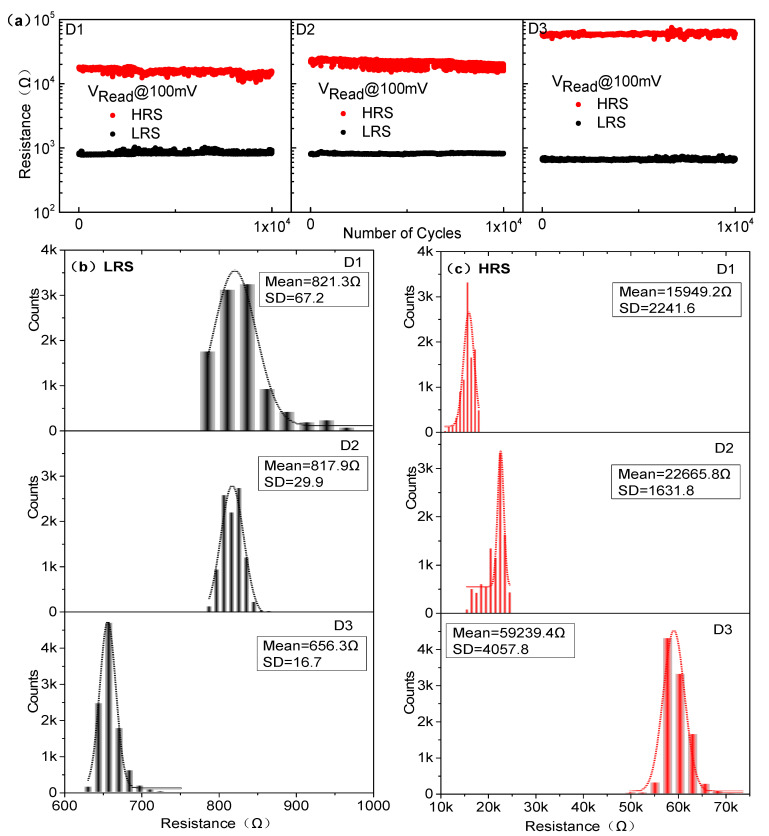
(**a**) Endurance of 10^4^ DC cycles of the D1, D2, and D3 devices, (**b**) Probability distribution histogram of (**b**) LRS and (**c**) HRS for the D1, D2, and D3 devices.

**Figure 5:**
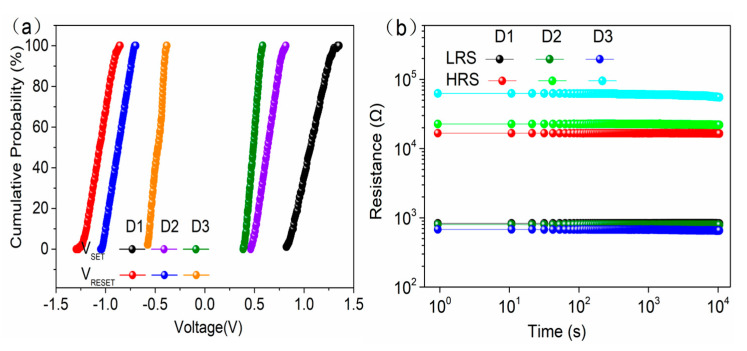
(**a**) Cumulative distributions of SET and RESET switching voltages for the D1, D2, and D3 devices, (**b**) Retention characteristics of the D1, D2, and D3 devices (V_Read_ at 100 mV for each device).

**Figure 6:**
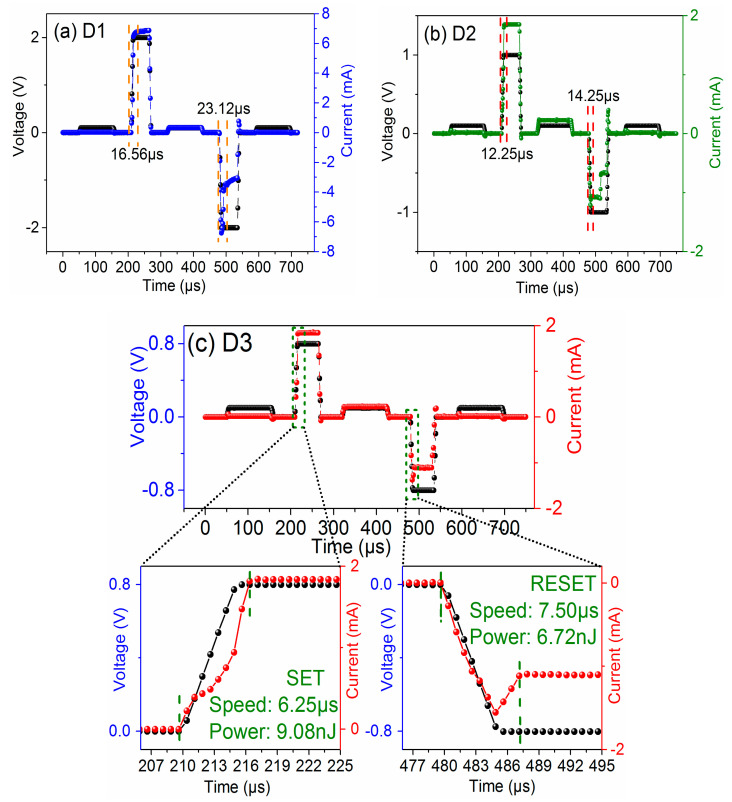
Switching speed of SET/RESET pulse pair for (**a**) D1, (**b**) D2, and (**c**) D3 devices. The black lines are the applied voltage pulses and the blue lines, green lines, and red lines are the separate current response pulses.

Figure 2a shows the typical bipolar resistive switching I–V curves of undoped and S-doped HfOx devices at an Icc of 1mA. The electrical forming process and the 10 continuous I–V cycles of each device are shown in Figure S1 of the Supplementary Materials. For ease of description, the undoped and S-doped 400 °C, 500 °C, and 600 °C devices are named D1, D2, D3, and D4, respectively. Compared with the D1, the RESET behavior of D2 and D3 is formed to be sharp gradually. More importantly, the Icc of the D3 device can be limited at 100 μA, as shown in Figure 2b, and the V_SET_/V_RESET_ of D3 is +0.11 V/−0.15 V, which will contribute to a low power property. In particular, the D2 device has a slight switching characteristic tendency at 100 μA, and D3 can only switch at 1 mA, as shown in Figure S2. The results show that S-doping plays an important role in the decrease in device switching current. Interestingly, the D4 device failed to exhibit the typical resistive behavior, as seen in detailed analysis of the Supplementary Materials. Meanwhile, in order to compare the effect of annealing treatment on the HfOx-based device, the sulfur undoped devices at 400 °C, 500 °C, and 600 °C were prepared and device performance was investigated, as shown in the Figure 2c. As a result, all of these devices show typical bipolar I–V characteristics, and compared with the untreated device, the device performance was changed to some extent. However, the device performance was not as significantly improved as that of the S-doped device, which indicates that S-doping plays a key role in improving device performance compared with the annealing treatment of HfOx-based RRAM. Figure 3 shows the AFM of the undoped and S-doped HfOx films. The root mean square (RMS) of the S-doped films was enlarged, which may be caused by the deposition rate of sulfur ions increasing with rising temperature, and increased the interface contact area between the ITO and the dielectric layer (ITO is a good oxygen storage layer [26]). As shown in Figure 4a, the HRS/LRS ratio increased obviously. Figure 4b,c shows probability distribution histograms of LRS and HRS, respectively. According to the Gaussian function, the statistical mean value and standard deviation (SD) are marked in the charts in Figure 4. The discrete coefficients (SD/mean ratio) of LRS (HRS) are 8.18%, 3.66%, and 2.54% (14.05%, 7.20%, and 6.85%) for D1, D2, and D3, respectively. A superior RS performance can be achieved with the D3 device. In addition, the endurance of 10^6^ alternate current (AC) cycles is shown in Figure S3 for D1, D2, and D3, respectively. For a comparison of the switching voltages, the cumulative distributions of V_SET_/V_RESET_ are presented in Figure 5a. According to the statistical voltage, the mean values of V_SET_ for D1, D2, and D3 are +1.05 V/+0.65 V/+0.51 V and SD are 0.095/0.051/0.012, respectively. The discrete coefficients of V_SET_ decreased from 9.05% to 2.35%. In addition, the mean values of V_RESET_ for three devices are −1.06V/−0.78V/−0.49V and the SD values are 0.086/0.056/0.013, respectively. The discrete coefficients decreased from 8.11% to 2.65%. S-doping reduced the V_SET_/V_RESET_ and improved the uniformity of the switching voltages. Figure 5b shows the retention characteristics of the S-doped device; the resistance values of both HRS and LRS exhibited robust stability and showed undetectable signs of degradation over 10^4^ s at 85 °C. Furthermore, in order to test the response speed of the devices, the SET/RESET pulses (width of 50 μs and amplitudes of ±2 V, ±1 V, ±0.8 V, respectively) were utilized to switch the resistance states, as illustrated in Figure 6a–c. The read pulses (100 μs/+0.1 V) were input before and after the SET/RESET pulses to monitor the resistance switching. The D3 could be switched to LRS (HRS) at a fast speed of 6.25 μs (7.50 μs) using the pulse pair (50 μs/±0.8 V), meanwhile, it consumes energy from the SET (RESET) process at 9.08 nJ (6.72 nJ). This can be calculated by using the formula:

W = V × I × T, (1)

where V is the applied voltage of the pulse, I is the response current, and T is the response time. In comparison, the SET/RESET speed of D1 is 16.56 μs/23.12 μs, and the energy is 224.2 nJ/155.8 nJ. A more explicit presentation of the D1 device is shown in Figure S4. S-doping is able to significantly improve the switching speed and achieve the low energy consumption in HfOx-based devices. Meanwhile, the resistive switching performance of undoped and S-doped HfOx-based devices are summarized in Table 1. The comparison of the effect of S-doping with other different doping elements for HfOx-based RRAM where their properties are simply summarized, is shown in Table S1.

**Figure 7:**
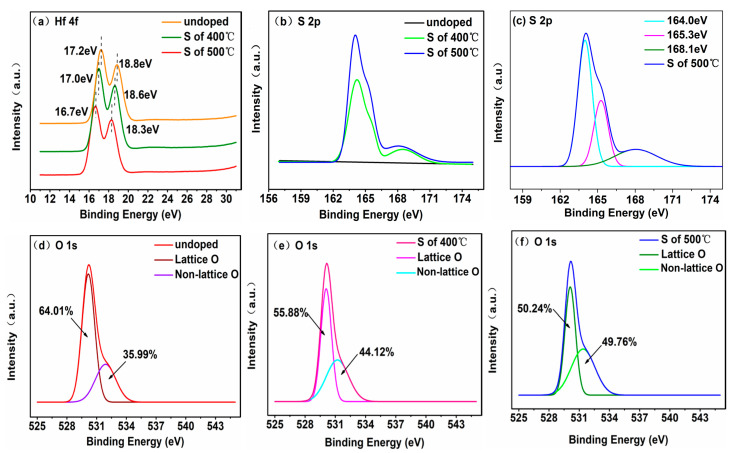
XPS spectra of (**a**) Hf 4f, (**b**,**c**) S 2p, (**d**–**f**) O 1s for the undoped and S-doped HfOx films.

**Figure 8:**
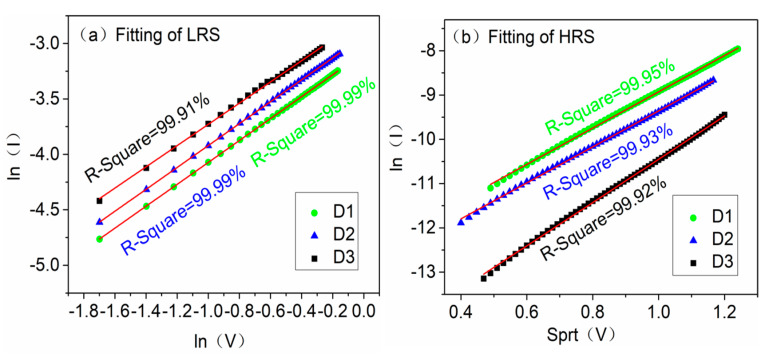
For the D1, D2, and D3 devices: (**a**) linear fit of LRS, (**b**) linear fit of HRS. (R-squared values are all greater than 99.9%, and are marked in the figure).

In order to clarify the effect of the S-doped HfOx device on the RS performance, the chemical composition of undoped and S-doped films was investigated using XPS. The binding energy was calibrated using the C–C bonds of an adventitious C signal (284.6 eV). Compared with the undoped film, the Hf 4f peaks of the S-doped film moved to a lower binding energy, indicating that sulfur ions incorporated into the HfOx films, as shown in Figure 7a. In Figure 7b, the S-doped films show a distinct peak of S 2p. XPS detected that the doping sulfur atomic percent content was 1.06 At.%/1.66 At.% under 400 °C/500 °C. The peaks fitting of S 2p spectra for S-doped of 500 °C HfOx film are shown in Figure 7c, and the binding energies of S 2p1/2 and S 2p3/2 are 165.3 eV and 164.0 eV, respectively, consistent with the S^2−^ valence state. According to the NIST X-ray Photoelectron Spectroscopy Database [27] and the shift of the Hf 4f peaks, indicating that there new bonds were formed in the S-doped HfOx film. Interestingly, there is a weak peak of binding energy at 168.1 eV and the corresponding valence state is S^4+^/S^6+^, which was caused by the impurity gas adsorbed onto the surface. Figure 7d–f shows the binding energies of the O 1s peaks for undoped and S-doped films. In the O 1s spectrum, the main peaks correspond to lattice oxygen at 529.8 eV, 530.0 eV, and 530.1 eV and the shoulder peaks correspond to non-lattice oxygen at 531.2 eV, 531.3 eV and 531.3 eV, respectively. Through the oxygen area percentages, the non-lattice oxygen components increased from 35.99% to 44.12% and 49.76%, indicating that oxygen vacancy contents increased after S-doping. The increased oxygen vacancies were more beneficial in forming and rupturing the CFs in the switching process [28,29]. The XPS spectra of Hf 4f, S 2p and O 1s for S-doped of 600 °C HfOx film are shown in Figure S5. It is worth mentioning that the non-lattice oxygen component in the S-doped 600 °C HfOx film was lower than that of the undoped film, which makes it harder for the D4 device to form oxygen vacancy type CFs. Afterwards, the conductive mechanisms were analyzed by the log-log linear fitting of LRS/HRS for D1, D2, and D3. Figure 8a shows the ln (I)–ln (V) linear fitting of LRS after SET. All the fitting slopes are ~1, indicating the Ohmic conduction mechanism in LRS. Figure 8b shows the ln (I)–sqrt (V) linear fitting of HRS after RESET, which proved to be consistent with the Schottky emission mechanism. Figure S6 shows that the Schottky emission mechanism was confirmed by variable temperature experiments.

**Figure 9:**
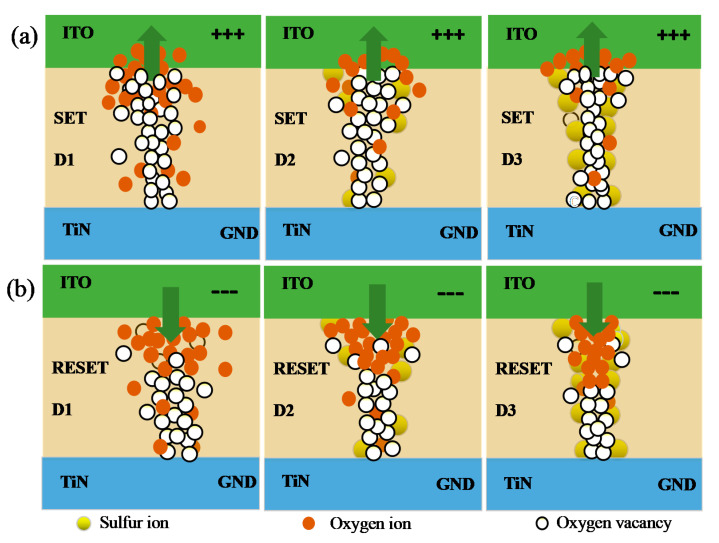
Schematic diagram of the switching mechanism of SET process for (**a**) D1, D2, and D3 and the RESET process for (**b**) D1, D2, and D3. (The green arrow represents the movement direction of oxygen ions, and the green box is the top electrode ITO of the device, and the blue box is the bottom electrode GND).

Based on XPS data and the fitting of the LRS/HRS conduction behavior, a switching mechanism was considered, as shown schematically in Figure 9a,b. The RS mechanism could be explained by the formation and rupturing of CFs, consistent with oxygen vacancies [30]. In the D1 device, the interstitial oxygen ions migrated toward TE under positive voltage. Moreover, the movement of oxygen ions was affected by joule heat and diffused around the film [31]. While TE was applied with a negative voltage, oxygen ions injected HfOx film from TE and combined with oxygen vacancies to break CFs. More frequently in the switching process, the partial oxygen ions break away from the modulation of the electric field and become unevenly distributed, which make the formed CFs unstable and random. The formation and rupture of CFs was more uniform in the D3 device. Oxygen ions moved in the same direction as the D1 device at positive and negative voltages. The lattice gaps of the HfOx film became larger because of the different bonding of Hf-S and Hf-O (the radius of the oxygen ion was 1.4 Å and that of the sulfur ion was 1.84 Å [32]), thus interstitial oxygen ions moved more easily in the gaps after S-doping. Furthermore, XPS proves that S-doping introduces more oxygen vacancies into HfOx film, which causes the S-doped HfOx film to be in a state of oxygen deficiency, and higher oxygen deficiency will favor a better performance for the device [31]. Furthermore, oxygen vacancies easily generate around S dopants to suppress the random formation of CFs [33,34], thus improving performance in the TiN/HfOx/ITO device.

## 4. Conclusions

In summary, S-doped HfOx RRAM devices were fabricated using APCVD treatment. The electrical performances are enhanced for the S-doped HfOx device, and show high uniformity, lower switching voltages, lager HRS/LRS ratio, stable endurance, long data retention characteristics, fast response speed, and low energy consumption of SET/RESET. Based on the experimental results, the improvements of the memory performance could be attributed to the S-doping effect in the HfOx-based RRAM. The results identify that S-doping increases oxygen vacancies in the HfOx-based device and could effectively suppress the randomness of oxygen vacancy CF formation. The results also demonstrate that the S-doped technology has potential applications in other oxide-based RRAMs.

## Figures and Tables

**Table 1:** Summarized the RS performance of undoped and S-doped HfOx-based devices.

Devices	V_forming_ (V)	Voltage (V)	HRS/LRS Ratio	Endurance (AC cycles)	Retention at 85 °C (s)	Switching Speed (μs)	Energy Consumption (nJ)
						SET/RESET	
D1	+2.18	+1.05/ −0.94	~12	10^6^ (Degradation)	10^4^	~16.56/~23.12	224.2/155.8
D2	+1.32	+0.76/ −0.78	~30	10^6^	10^4^	~12.25/~14.25	22.54/15.25
D3	+0.85	+0.11/ −0.15	~90	10^6^ (Uniformity)	10^4^ (Uniformity)	~6.25/~7.50	9.08/6.72
D4	-	+2/−2	~2	-	-	-	-

## Data Availability

Data available on request. The data presented in this study are available on request from the corresponding author.

## Supplementary Materials

The following are available online at https://www.mdpi.com/article/10.3390/ma14123330/s1, Figure S1: The forming process of (**a**) D1, (**b**) D2, (**c**) D3, (**d**) D4 and the consecutive I–V cycles of (**e**) D1, (**f**) D2, (**g**) D3, respectively. Figure S2: The I–V curves of each device under 100 μA and 1 mA compliance current. Figure S3: The endurance of 10^6^ AC cycles for D1, D2, D3 devices, respectively. Figure S4: The switching speed upon SET/RESET pulse pair for (**a**) D1 and (**b**) D2. Figure S5: XPS spectra of (**a**) Hf 4f, (**b**) S 2p, (**c**) O 1s for S--doped of 600 °C HfOx film. Figure S6: The Schottky emission fitting for (**a**) D1, (**b**) D2, (**c**) D3 devices at temperature ranged from 300 K to 340 K. Table S1: Summary of the properties of HfOx-based RRAM for different doping elements.
