# Supplementary material for: Improvement of Resistive Switching Performance in Sulfur-Doped HfOx-Based RRAM
Source: Materials (Basel). 2021 Jun 16;14(12):3330. doi: 10.3390/ma14123330 (PMC8233777; doi:10.3390/ma14123330)
Supplement: Supplementary file 1 [file materials-14-03330-s001.zip › materials-1227320-supplementary.pdf]

materials

MDPI

Supplementary Material

Improvement of Resistive Switching Performance in Sulfur-Doped HfOx-Based RRAM

Zhenzhong Zhang [1], Fang Wang [1], Kai Hu [1], Yu She [1], Sannian Song [2], Zhitang Song [2] and Kailiang Zhang [1,*]

1. Tianjin Key Laboratory of Film Electronic and Communication Devices, School of Electrical and Electronic Engineering, Tianjin University of Technology, Tianjin 300384, China.
2. State Key Laboratory of Functional Materials for Informatics, Shanghai Institute of Micro-System and Information Technology, Chinese Academy of Sciences, Shanghai 200050, China.

The forming processes of D1, D2, and D3 devices are shown in Figure S1a–c, which are the typical positive forming processes. Due to the introduction of oxygen vacancies into HfOx films after S-doping, the forming voltages ($V_{forming}$) of D2 and D3 are reduced to +1.32 V and +0.85 V, compared with the undoped D1 device ($V_{forming}$ is +2.18 V). The small $V_{forming}$ has little effect on the subsequent SET/RESET switching process, which is beneficial to reduce the switching voltages of S-doped devices. In the experiment, even if the stimulating voltage is increased to +10 V or −10 V, the D4 device still does not have obvious resistance state switching, as shown in Figure S1d, and it has lost its excellent characteristics as a resistive memory. In the forming process, the Icc of D1, D2, and D3 are uniformly set to 1 mA. The 10 consecutive I–V cycles of D1, D2, and D3 devices are shown in Figure S1e–g. The I–V curves of each device under 100 μA and 1 mA compliance current are shown in Figure S2. In addition, the endurance of 10^6 alternate current (AC) cycles is shown in Figure S3 for D1, D2, and D3 devices, respectively.

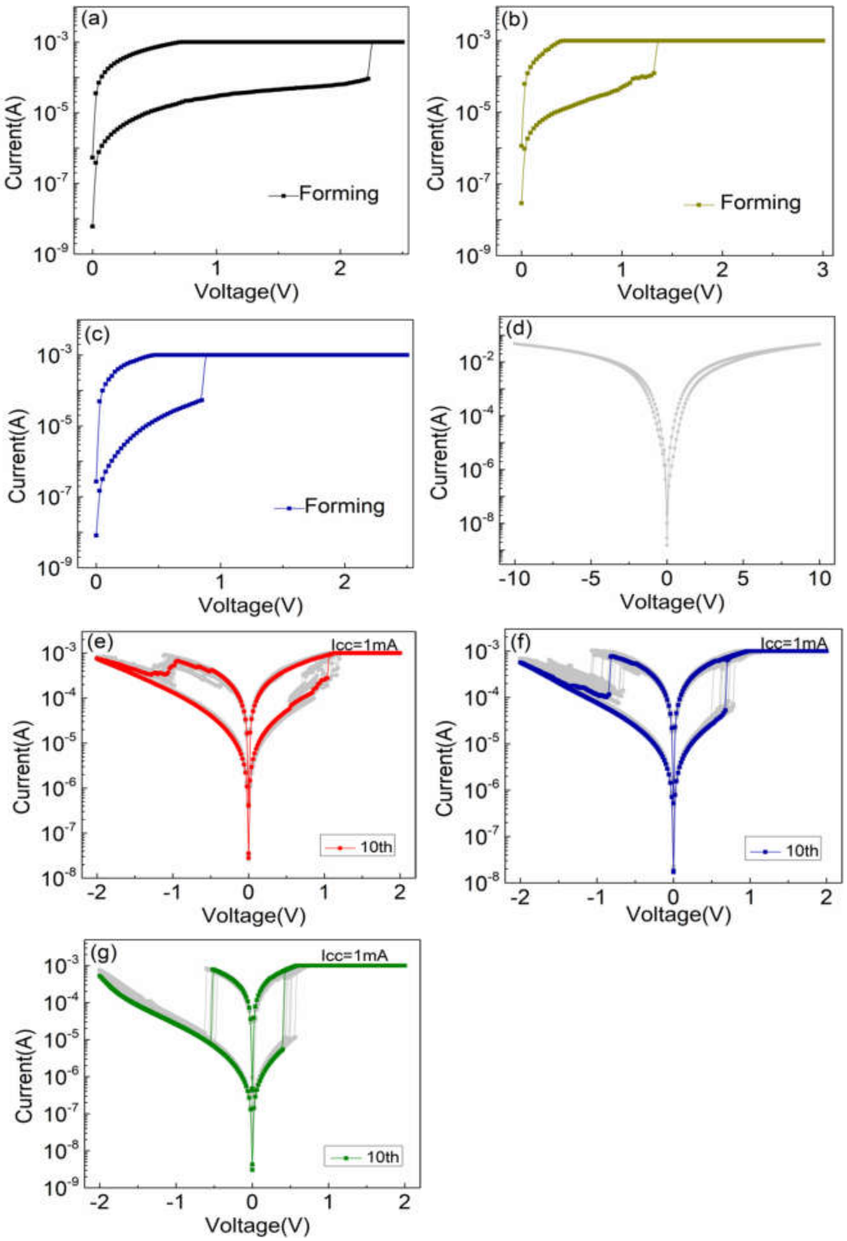

Copyright: © 2021 by the authors. Licensee MDPI, Basel, Switzerland. This article is an open access article distributed under the terms and conditions of the Creative Commons Attribution (CC BY) license (http://creativecommons.org/licenses/by/4.0/).

Figure S1. The forming process of (**a**) D1, (**b**) D2, (**c**) D3, and (**d**) D4 and the consecutive I–V cycles of (**e**) D1, (**f**) D2, and (**g**) D3, respectively.

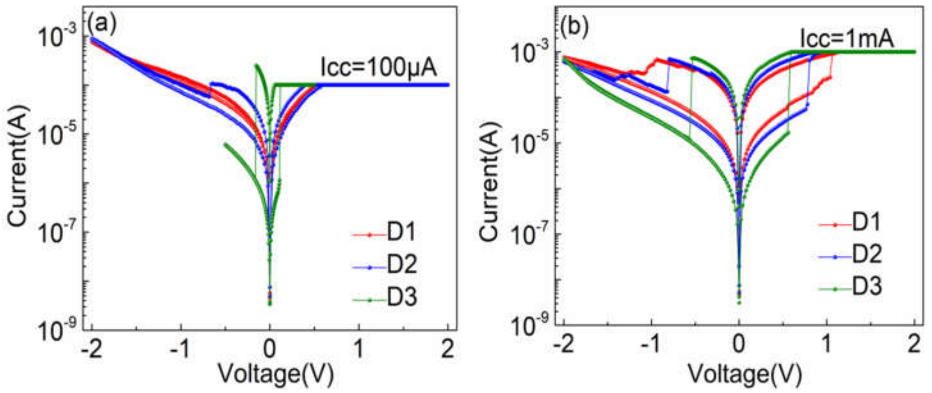

Figure S2. The I–V curves of each device under 100 μA (**a**) and 1 mA compliance current (**b**).

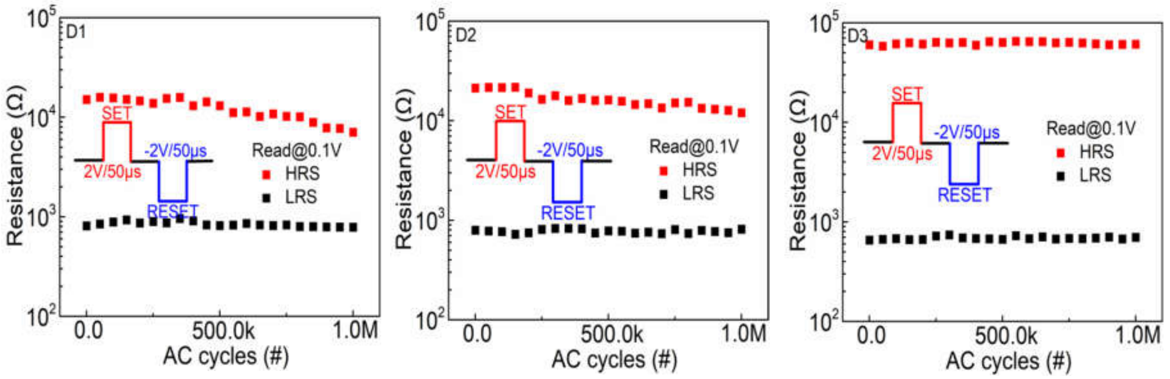

Figure S3. The endurance of 10^6 AC cycles for D1, D2, and D3 devices, respectively.

Figure S4 shows a more intuitive resistance state switching of the D1 and D2 devices under the applied pulse pair. The SET/RESET switching speed of D1 (D2) is 16.56 μs/23.12 μs (12.25 μs/14.25 μs), and the energy is 224.2 nJ/155.8 nJ (22.54 nJ/15.25 nJ). Under the appropriate sulfur-doping content, the SET/RESET switching speed and the energy consumption of D3 device are 6.25 μs/7.50 μs and 9.08 nJ/6.72 nJ. The switching speed is improved and the energy consumption is reduced after S-doping.

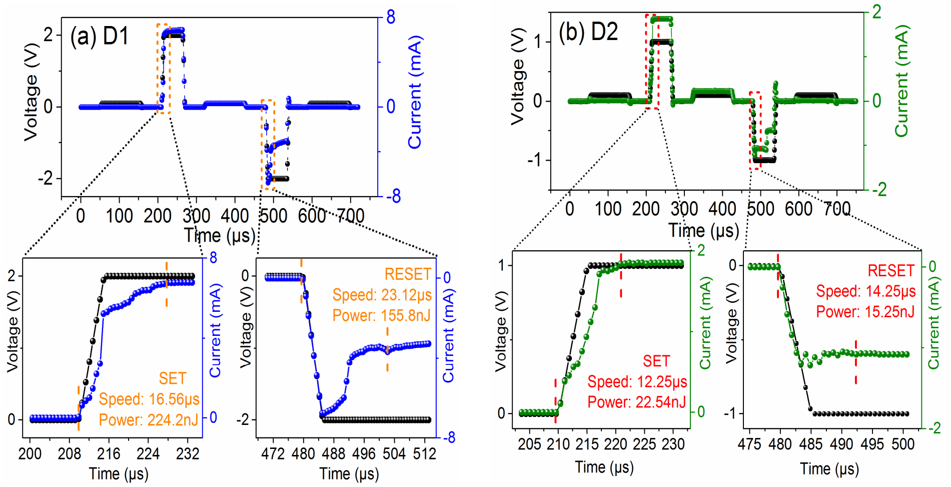

Figure S4. The switching speed upon SET/RESET pulse pair for (**a**) D1 and (**b**) D2.

The XPS spectra of Hf 4f, S 2p and O 1s for S-doped of 600 °C HfOx film are shown in Figure S5. The binding energy of Hf 4f (Hf 4f 7/2 at 16.3 ev and Hf 4f 5/2 at 17.9 ev) is

lower than that of the undoped HfOx film, which corresponds to the binding energy of Hf 4f in HfS_2 [6]. In addition, the atomic ratio of sulfur to oxygen to hafnium (S:O:Hf) inside the film is calculated to be about 1.2:0.8:1 by the ratio peak area and sensitivity factor of each element in XPS, which suggests that the bonding of Hf-S more than the Hf-O. And the non-lattice oxygen component in the S-doped of 600 °C HfOx film is lower than that of the undoped film, which makes it harder for the D4 device to form oxygen vacancy type CFs.

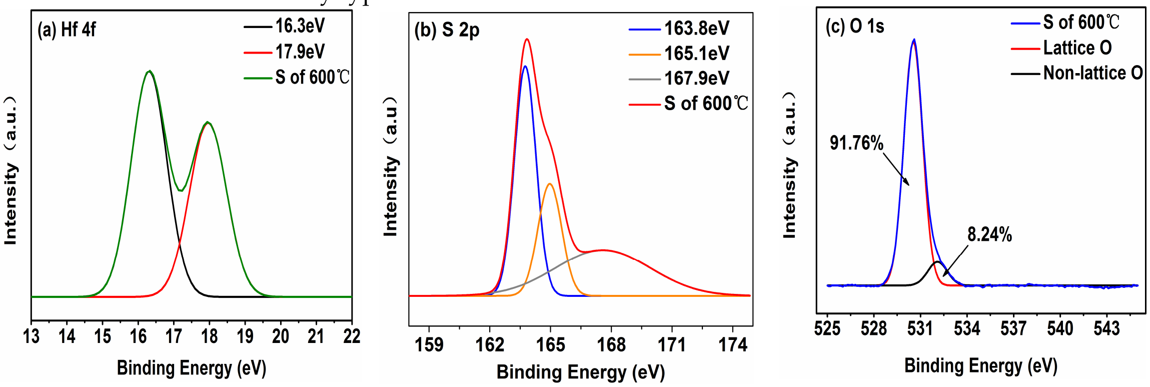

Figure S5. XPS spectra of (**a**) Hf 4f, (**b**) S 2p, (**c**) O 1s for S--doped of 600 °C HfOx film.

In order to further verify the Schottky emission mechanism, the temperature effect experiment is done in the temperature range of 300–340 K, as shown in Figure S6a–c for D1, D2, and D3 pristine devices, and the curves show obvious temperature dependence in the absolute higher-voltage region.

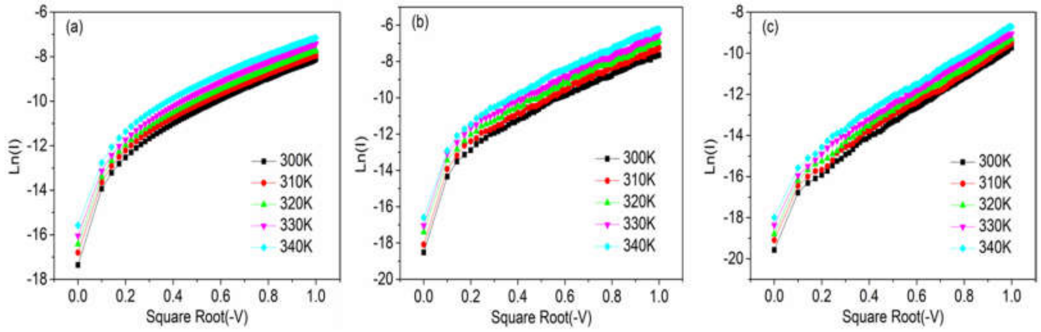

Figure S6. The Schottky emission fitting for (**a**) D1, (**b**) D2, and (**c**) D3 devices at temperature ranged from 300 K to 340 K.

Table S1. Summary of the properties of HfOx-based RRAM for different doping elements.

Doping Elements		Device Structure (BE/Insulator/TE)	Impact on the Device	$V_{forming}$ (V)	Icc (A)	Switching Voltage(V)	Endurance	Retention	Switching Speed (SET/RESET)	Energy Consumption	References
Metal	Al	Pt/HfOx/Cu	Oxygen vacancies easily generated along Al dopants	+0.8	10 m	+0.3/−0.45	10^2 cycles @ DC	10^4s @25 °C	-	-	[1]
	Cu	Pt/HfO_2/Cu	Cu conducting filaments	-	1 m	+3/−1.8	10^2 cycles @ DC	10^5s @25 °C	10 ns/100 µs	-	[2]
	Ni	Pt/HfOx/Au	The reduction of the activation energy of oxygen vacancies	+1.8	1 m	+0.16/−0.18	10^2 cycles @DC	10^4s @25 °C	-	-	[3]
Non-metal	N	TiN/HfO_2/Pt	Deep defect states exist after nitridation treatment	+5.9	100 µ	+0.7/−0.9	10^6 cycles @ AC	10^4 s @85°C	-	-	[4]
	N	Pt/Ti/HfO_2/Pt	N doping can effectively suppress the randomicity of filaments formation	+3.4	10 µ	+1.14/−1.02	10^3 cycles @DC	10^5 s @25 °C	-	-	[5]
	S	TiN/HfOx/ITO	More oxygen vacancies are introduced into film by the S dopants	+0.85	100 µ	+0.11/−0.15	10^6 cycles @AC	10^4 s @85 °C	6.25 µs/7.5 µs	6.72 nJ	This work

References:

1. Guo, T.; Tan, T.; Liu, Z.; Liu, B. Oxygen vacancy modulation and enhanced switching behavior in HfO x film induced by Al doping effect. *J. Alloy. Compd.* **2016**, *686*, 669–674, doi:10.1016/j.jallcom.2016.06.090.
2. Wang, Y.; Liu, Q.; Long, S.; Wang, W.; Wang, Q.; Zhang, M.; Zhang, S.; Li, Y.; Zuo, Q.; Yang, J.; et al. Investigation of resistive switching in Cu-doped HfO2thin film for multilevel non-volatile memory applications. *Nanotechnol.* **2009**, *21*, 045202, doi:10.1088/0957-4484/21/4/045202.
3. Tan, T.; Du, Y.; Cao, A.; Sun, Y.; Zha, G.; Lei, H.; Zheng, X. The resistive switching characteristics of Ni-doped HfO film and its application as a synapse. *J. Alloy. Compd.* **2018**, *766*, 918–924, doi:10.1016/j.jallcom.2018.07.044.
4. Wang, M.-H.; Chang, T.-C.; Shih, C.-C.; Tseng, Y.-T.; Tsai, T.-M.; Zheng, H.-X.; Wu, P.-Y.; Huang, H.-C.; Chen, W.-C.; Huang, J.-W.; et al. Performance improvement after nitridation treatment in HfO2-based resistance random-access memory. *Appl. Phys. Express* **2018**, *11*, 084101, doi:10.7567/apex.11.084101.
5. . Xie, H.; Liu, Q.; Li, Y.; Lv, H.; Wang, M.; Liu, X.; Sun, H.; Yang, X.; Long, S.; Liu, S.; et al. Nitrogen-induced improvement of resistive switching uniformity in a HfO2-based RRAM device. *Semicond. Sci. Technol.* **2012**, *27*, 125008, doi:10.1088/0268-1242/27/12/125008.
6. Yan, C.; Gan, L.; Zhou, X.; Guo, J.; Huang, W.; Huang, J.; Jin, B.; Xiong, J.; Zhai, T.; Li, Y. Space-Confined Chemical Vapor Deposition Synthesis of Ultrathin HfS2 Flakes for Optoelectronic Application. *Adv. Funct. Mater.* **2017**, *27*, 1702918, doi:10.1002/adfm.201702918.
